# Understanding a videogame home intervention for children with hemiplegia: a mixed methods multi-case study

**DOI:** 10.3389/fmedt.2023.1217797

**Published:** 2023-07-12

**Authors:** Daniela Chan-Víquez, Ajmal Khan, Sarah Munce, Darcy Fehlings, F. Virginia Wright, Elaine Biddiss

**Affiliations:** ^1^ Bloorview Research Institute, Holland Bloorview Kids Rehabilitation Hospital, Toronto, ON, Canada; ^2^ Rehabilitation Sciences Institute, University of Toronto, Toronto, ON, Canada; ^3^ KITE-Toronto Rehabilitation Institute, University Health Network, Toronto, ON, Canada; ^4^ Department of Paediatrics, Temerty Faculty of Medicine, University of Toronto, Toronto, ON, Canada; ^5^ Institute of Biomedical Engineering, University of Toronto, Toronto, ON, Canada

**Keywords:** cerebral palsy, hemiplegia, exergaming, telerehabilitation, motivation, augmented reality, mixed methods

## Abstract

**Introduction:**

Access to rehabilitation therapies is a salient and growing issue for children with cerebral palsy (CP) and their families, motivating interest in home-based interventions. Bootle Blast is a low-cost, movement-tracking videogame that can be used at home to encourage upper limb (UL) functional exercise tailored to each child's abilities and therapy goals. The study objectives were to: 1) Establish the extent to which children achieve their self-directed play-time goal over a 12-week intervention, 2) Measure changes in UL motor outcomes, and 3) Explore participants' experiences of using Bootle Blast at home.

**Methods:**

Mixed methods case series study of four children with hemiplegic cerebral palsy (HCP), each with a participating parent. Participants played Bootle Blast at home for 12 weeks. Study assessments occurred at baseline, post-intervention and four week follow up. A post-intervention interview explored participants' experiences. Game-logs provided play time and progress data.

**Results:**

Three of four participants (8-13 yrs., Manual Ability Classification Level I-II) completed the intervention. One dropped out at week 6. Play-time goals were achieved in most weeks, with two of four children surpassing their overall intervention goals. Outcomes varied across the three participants, however consistent improvements were observed on the Canadian Occupational Performance Measure and the Box and Blocks Test. Inductive analysis generated four main themes: 1) Intrinsic motivators fostered play engagement, 2) Virtual play for real-world gains, 3) Therapy on demand (at home), and 4) Shifting the onus from the parent to the game. Integration of qualitative and quantitative data was important for interpreting play patterns/usage and clinical outcomes.

**Discussion:**

This mixed methods study describes a novel videogaming intervention designed for home-rehabilitation for children with HCP and provides preliminary evidence to guide future study design and research.

**Clinical Trial Registration:**

[https://clinicaltrials.gov/ct2/show/NCT04009031?recrs=h&cond=Cerebral+Palsy&cntry=CA&city=Toronto&draw=2&rank=1], identifier [NCT04009031].

## 1. Introduction

Cerebral palsy (CP) is the most common neuromotor childhood disability, affecting around 2.11 children per 1,000 live births in developed countries. It is a permanent disorder that impacts the development of movement and posture (1). Between 60% and 83% of children with CP have upper limb (UL) involvement (2). Hemiplegic or unilateral CP is the most common sub-type (approximately 38%), affecting one side of the body (3). Children with hemiplegia often exhibit developmental disregard—a learned behavior which manifests in suppressed use of the hemiplegic UL in activities of daily living in a way that is mismatched with its capacity (4). This can lead to delays in the development of fine motor skills and reduced independence in completing bimanual activities. This phenomenon can have significant implications for the child’s quality of life and functional independence and is addressed through therapeutic strategies like constraint induced movement therapy that offer enriched opportunities to use the hemiplegic UL, thereby reducing the suppression of motor activity (4, 5).

Rehabilitation therapies that are intensive and goal-oriented can improve UL motor skills, disregard and function (6). However, consistent access to clinician-directed services can be limited due to economic and geographic constraints (7). Home-based rehabilitation aims to increase the amount and frequency of therapy practice for children with CP. However, adherence to traditional home programs can be negatively impacted by limited time to incorporate the program into daily routines and challenges in motivating repetitive practice (8, 9). Alternative novel play-based approaches that are built on therapeutic principles are needed to support children with CP in engaging in these home programs. In the last decade, movement tracking videogames have been introduced and used to engage children with disabilities in home-based therapy. Commercially available systems (e.g., Nintendo Switch) are lower-cost and accessible but are not configurable to the child’s abilities and therapy goals. Systems designed specifically for therapeutic use are more effective in improving UL motor outcomes, however sustaining engagement over extended periods of time and supporting access to specialized equipment in the home remains a challenge (10–12). Additional challenges to successfully integrating videogames into home-based therapy include ensuring quality of movements and system configuration (13).

In an effort to balance the advantages of entertainment gaming systems and the therapeutic value of engineered rehabilitation systems, an interdisciplinary team (i.e., researchers, physical and occupational therapists, physicians, engineers, game designers/developers, user experience specialists, composers, digital artists/animators) at Holland Bloorview Kids Rehabilitation Hospital (herein referred to as, Holland Bloorview) worked with children with diverse abilities and diagnoses including CP, their siblings and caregivers, to design the Bootle Blast (BB) videogame system. BB was developed in an iterative co-creation process spanning 8 years. BB targets a range of upper body therapy goals (e.g., cross-body reach, shoulder abduction/adduction, grasp and release) in 13 mini-games (Supplementary Appendix A) linked by an overarching narrative and reward system. Movements of the body are detected via a 3D camera (Microscoft Kinect 2) and associated skeletal tracking software. Five of the mini games support the development of fine motor skills via mixed-reality wherein the player manipulates real-life objects (e.g., MegaBloks, musical instruments like maracas) during gameplay. Manipulation of these objects is tracked through a combination of depth sensing, color tracking and audio detection (i.e., for musical instruments). A short video demonstrating the game is available at https://youtu.be/jxvCxh7mudE.

Each mini-game can be calibrated to each child’s range of movement for their dominant and non-dominant arm (via a “window washing” game) and therapy goals (via menu settings). The game can be played in timed or life mode. In timed mode, games are played for a set period of time regardless of performance. In contrast, life mode offers a greater challenge as the ability to progress or continue to play in the game is tied to the player’s performance. In both modes, the game dynamically scales in difficulty (e.g., speed of movements required) as children practice and achieve different levels. Eight out of 13 mini-games have a two-player mode to encourage social play. Lastly, there is a reward system in BB that is highly linked to achieving an individualized play time goal (PTG)—the time per week that the individual chooses to play (minutes per session, sessions per week)—over the course of the intervention. Of note, there is no consensus as to what an effective “therapeutic dose” is for interactive computer play-based therapy nor how to measure it (12). The “effective” dose likely depends on specific attributes of the gaming system (e.g., intensity of the game) as well as the child (e.g., motor skills, therapy needs) (14). BB’s customizable PTG enables the therapy program to be adapted to the child’s capacity and to family dynamics/daily schedule, positioning the child for success. This PTG is based on active game play only and excludes passive game play (e.g., time spent navigating game menus, perusing high scores). BB has a tiered reward system with immediate rewards provided for exercises completed in the mini-games (e.g., scores, visual feedback), mid-term rewards for achieving the daily PTG (e.g., coins, achieving new levels—“leveling up”), and longer-term rewards linked to sustained use of the system over the course of the intervention (e.g., completing the game mission of collecting 100 rare Bootle robots) (15).

The purpose of this study was to investigate the use of BB for UL home-based rehabilitation for children with HCP over 12 continuous weeks. Our objectives were to: (1) establish the extent to which children achieve their weekly play-time goal (PTG) over a 12-week intervention (adherence), (2) measure changes in UL motor outcomes, and (3) explore participants’ experiences of using BB for home rehabilitation. A clinic version of BB has been used at Holland Bloorview since October 2017 in over 1,000 on-site therapy sessions with children with CP, acquired brain injury, spinal cord injury, and during recovery from orthopedic surgeries, however this is the first research study reporting on the use of BB.

## 2. Methods

### 2.1. Study design, paradigm, and conceptual framework

**Figure 1:**
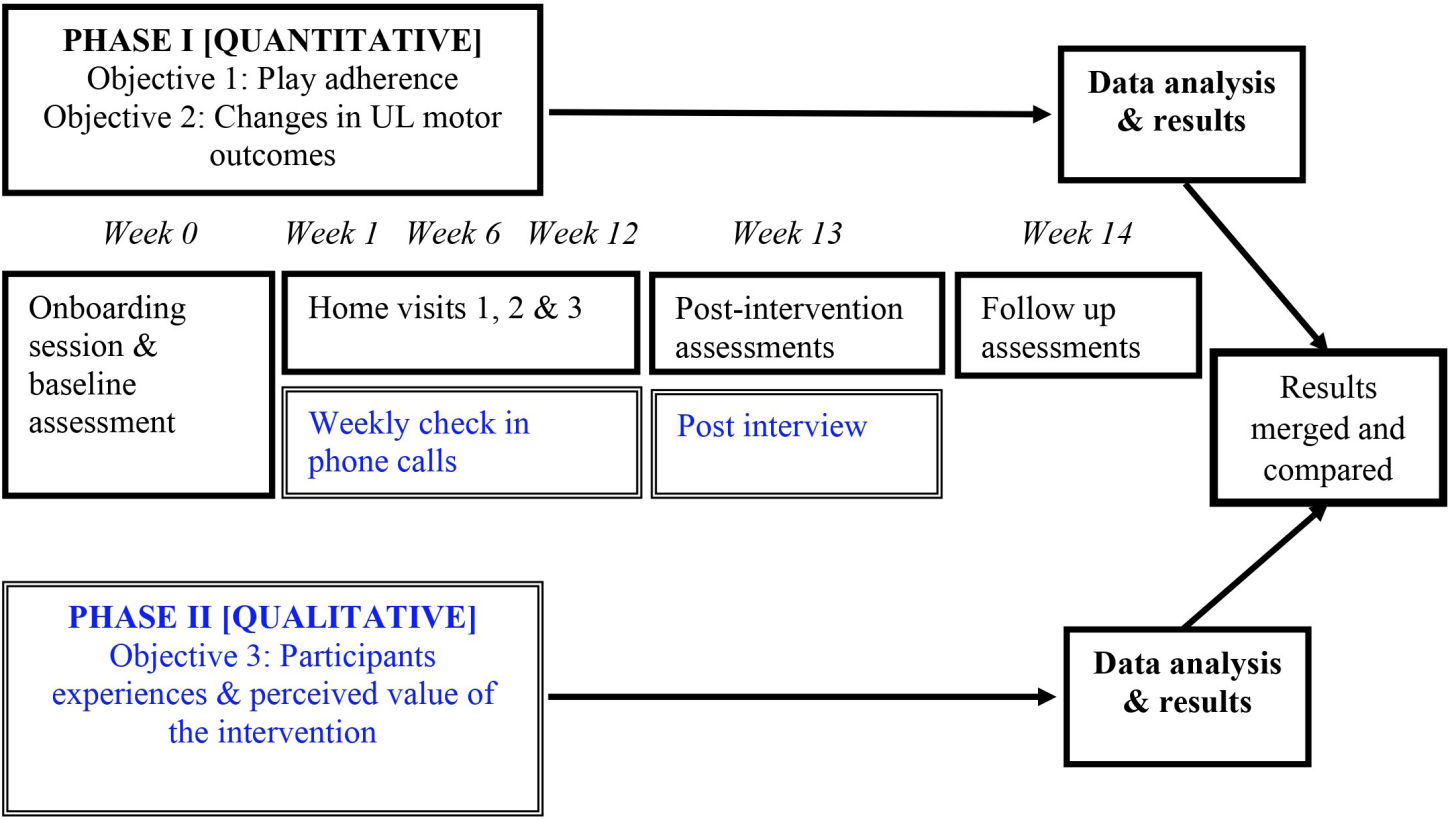
Multi-case mixed methods study design, data collection timeline and integration of phases. qualitative measures (double line) are indicated in blue and quantitative measures (solid) in black.

A multi-case, mixed methods was used to develop an in-depth understanding of the family’s experience with BB, explore how/if quantitative outcomes (e.g., UL motor outcomes, adherence) complement/differ from the lived experiences described in the interviews, and support comparisons across the cases (16) (children from different cultural backgrounds and living contexts in Toronto, Ontario, Canada). In Phase 1, the enrolled child-parent dyads were loaned BB to play at home for 12 continuous weeks, and participated in three clinical research assessments (pre, post and four weeks post follow up). Phase 1 used a prospective interventional design and evaluated pre-post changes in UL motor outcomes and trends in play adherence. During the 12-week intervention, a monitoring therapist performed weekly check-in phone calls with participants. Phase 2 used a qualitative descriptive approach (17) and explored the participants’ experiences of using BB for home rehabilitation through semi-structured interviews with children and caregivers. Figure 1 summarizes the two-phase study design and data collection protocol.

This study is situated within the paradigm of pragmatism and enables bridging of our clinical experience and research views to better understand the BB experience (18). A patient and family-centered approach framework was used to better tailor and understand the results of the intervention. This framework supports interventions and practices where families are encouraged to make informed decisions and the client’s strengths are prioritized. The patient and family-centered framework also encourages a collaborative family and health provider relationship (19). Quantitative and qualitative data were given equal priority, analyzed separately and then brought together for interpretation (see Integration section). This manuscript follows the Case Report Guideline (20) and Good Reporting of a Mixed Methods Study (19) guidelines for case report and mixed methods, respectively. Ethical approvals were obtained from Holland Bloorview and the University of Toronto. The study was registered on clinicaltrials.gov (NCT04009031).

### 2.2. Phase 1 (quantitative): play adherence and changes in UL motor outcomes

#### 2.2.1. Participants

A volunteer sample was recruited from Holland Bloorview’s Connect2Research database. Eligible participants were called and the first four child-parent dyads who gave informed assent/consent were enrolled. A sample size of 4 to 5 participants is considered appropriate for mixed methods case study research (16). With respect to eligibility, all children needed to be 7–17 years old, have a diagnosis of HCP with involvement of the UL, and normal or corrected to normal vision and hearing. Children could not have received constraint induced movement therapy in the past six months, or botulinum toxin injections or active UL therapy within three months of study enrolment.

#### 2.2.2. Protocol

##### 2.2.2.1. Baseline assessment and onboarding

At baseline, children completed a 45min-to-1 h clinical research assessment with an occupational therapist (OT) followed by a 30 min onboarding session with BB at Holland Bloorview. The same OT completed all clinical assessments and was blinded to previous scores. During the onboarding, BB was customized to the child’s therapy goals (i.e., mini-games targeting the exercises/movements of interest were selected). Additionally, BB was calibrated to the child’s range of movement and reach via a window-washing game. Following this, the child played a selection of mini-games allowing the research team (DC [pediatric physical therapist with experience working with families and young people with CP], and AK [BB software engineer]) to confirm that the child could play and understand the games. DC identified if any modifications or supports were needed (e.g., caregiver support while playing, adapting mixed-reality objects to achieve proper grasp). Finally, the child and caregiver were instructed on how to navigate and use BB at home.

##### 2.2.2.2. Home intervention

Within one week of the baseline assessment, two researchers (DC, AK) visited the child’s home to deliver and set up the videogame system which consisted of:

• A laptop with BB and the 3D camera required to play it (Microsoft Kinect®).
• A box with toy instruments (a maraca or a small egg-shaped shaker, a tambourine, a xylophone, and a hand-held castanet, Rhythm Band Instruments, TX, USA) and coloured building blocks (Mega Bloks, Mattel, CA USA) to play the mixed-reality games.
• A user manual with explanations, pictures, play and troubleshooting tips.

During this home visit, DC worked with the child and caregiver to identify a feasible weekly PTG for the family (i.e., minutes per day, days per week) considering their schedule and the child’s preferences. Children played BB at home for 12 consecutive weeks, with home visits at weeks six [to complete the Box and Block test, BBT (22)] and 12 (to pick up the system). During the 12-week intervention, DC (in a monitoring therapist role) called every week (5–10 mins) to answer questions, troubleshoot/document any technical problems, and gain an understanding of the participants’ experience during that week. Field notes (23) were taken during each of these conversations.

##### 2.2.2.3. Post-assessment and follow-up assessments

Participants completed clinical assessments at Holland Bloorview within one week post-intervention, and a follow-up assessment was conducted four weeks later. Outcome measures were as follows:

*Canadian Occupational Performance Measure (COPM)* evaluates self- or parent-reported satisfaction and performance on self-identified therapy goals. Participants were asked to identify one to three hand/arm goals associated with ADLs that they wished to improve (e.g., cutting with fork and knife). Parent and child rated together (when possible) the child’s level of performance and satisfaction with performance on a 10-point scale (1 is poor/low and 10 is good/high) for each of the identified goals. The COPM has good reliability, construct validity and responsiveness for use with children with CP (24, 25).

*Assisting Hand Assessment (AHA)* evaluates the use of the affected hand in assisting during the performance of 22 bimanual activities using objects in the AHA toy kit. Each task is rated on a 4-point rating scale (4 = effective, 0 = does not do). Tasks involve object manipulation which is scored under the categories of general use, arm use, grasp and release, fine motor adjustments and coordination and pace. Rasch analysis converts raw scores into a logit-based scale ranging from 0 to 100, with higher scores representing a higher ability. This measure has shown high test-retest reliability, validity and responsiveness for children with CP (26, 27).

Secondary outcome measures consisted of the following: I) active range of motion (aROM) of shoulder, elbow and wrist joints (28) ii) hand grip strength using a modified sphygmomanometer (child sitting, elbow flexed at 90°, shoulder abducted and wrist as close as possible to the neutral position) (29); the BBT to measure unilateral gross manual dexterity (22, 30) and the parent-report Children’s Hand Use Experience Questionnaire (CHEQ) to capture perceived quality and effectiveness of the child’s use of their affected hand in bilateral task performance. The CHEQ is scored in a unit scale from 0 (worse/lowest score) to 100 (better/max score). Additionally, it provides a count on the number of activities the child is able to perform bimanually, with help or with one hand (out of 27 bimanual activities) (31, 32).

Other data collected during the home intervention consisted of:

• Computer logs consisting of date/time of play sessions, active play time, game performance (scores) and progress (levels) were logged to determine adherence.
• Monitoring therapist’s notes: to outline the duration and content of communications (including technical assistance requests), and therapist’s views on the experiences, including challenges, of BB faced by the parent and child.

#### 2.2.3. Data analysis

To address objective 1, we calculated the extent to which children met their weekly PTG, and in how many weeks this goal was achieved. For Phase 1, journal field notes from the weekly monitoring phone calls with DC documented technical barriers, as well as each participant’s lived experience with BB during each week of the intervention. All functional measures were assessed and scored by the study’s OT assessor at the end of each assessment session. To limit bias, the assessor was not involved in the intervention. To address objective 2, clinical assessment scores were visually compared at baseline, post-intervention and follow up for each participant. Results were interpreted based on their minimally clinical important difference (MCID) when available. Specifically, for the COPM, a change of 2 points is considered a MCID (25). Smallest detectable change and MCIDs are not yet available for the CHEQ; however, previous studies have considered a 10% positive change in percent of bimanual activities performed independently to be clinically important for children with CP (33). A MCID for aROM of 10 degrees was used. The mean grip strength from three successive trials was used, and a MCID of at least 10 mm Hg was considered (34). In absence of MCID estimates, the smallest detectable change (SDC) was applied for the AHA [i.e., 5 logit units (27)] and BBT [i.e., 2 blocks in the more affected hand (30)].

### 2.3. Phase 2 (qualitative): participants’ experiences and perceived value of the intervention

#### 2.3.1. Data collection

The in-person interviews with the parents and children in Phase 1, were done separately immediately following the post-intervention clinical assessment. Two researchers conducted the interviews (AH for 2 dyads, and BR for 1 dyad). Both interviewers were male, part of the BB research and development team, had experience working with families and children with disabilities and experience conducting interviews. Neither had met the participants before the interview. The participants were not made aware of the involvement of the interviewer with BB in order to limit any hesitation in expressing concerns or negative experiences that they may have had with BB.

The monitoring therapist (DC) met with the interviewer beforehand to discuss the participant dyad and provide an overview of the family’s experiences with BB over the 12-week intervention (e.g., technical barriers experienced, changes/modifications in the program made). Each parent and child interview lasted between 20 and 30 min and was audio-recorded. Semi-structured, open-ended questions (Supplementary Appendix B) explored participants’ experiences (e.g., enjoyment of the videogame/therapy mode), perceived value (e.g., usefulness of the intervention for hand/arm therapy), and motivational factors facilitating adherence to the intervention, as well as their perspectives on identifying their own PTGs.

#### 2.3.2. Data analysis

To address objective 3, verbatim transcripts from interviews were independently coded by DC (pediatric physiotherapist and PhD student), a research assistant (RA, master’s in occupational therapy student), and one of the co-authors (EB, primary investigator with over 15 years of experience conducting childhood disability research). All coders identified as female. Interviews were analyzed using a descriptive, inductive thematic approach (17). NVivo software was used to manage the data. Initial interpretations of the data were discussed by DC and EB, and codes developed based on emerging themes from participants’ responses. Text segments were assigned codes, and then grouped into potential themes and sub-themes to show patterns in participant responses. Participant characteristics (e.g., socio-economic status, age) were considered to understand the context of the individual’s experiences and perspectives. Code-recode, peer examination and field/reflexive notes were used to ensure qualitative rigour (35).

#### 2.3.3. Integration

Integration of the quantitative and qualitative strands occurred at the methods, results and interpretation levels. Team members (DC, EB, FVW, SM) involved in the integration process had a (clinical) background in childhood disability, quantitative, qualitative, and/or mixed methods expertise. At the methods level, a nested sample from phase 1 was used in phase 2 (i.e., connecting, all participants who completed Phase 1 were included in Phase 2). Additionally, data from the weekly phone calls informed the post intervention interview guide (i.e., building) (36). For example, parents were asked about the main barriers they faced when weekly PTGs were not met, and children were asked to further comment on game features, and how/if they helped them in achieving their PTGs. Data sets were analyzed separately first and then merged and compared for interpretation to address our research objectives (Figure 1). In the results below, a narrative, weaving approach has been used whereby qualitative and quantitative results are presented together for each dyad and organized according to the themes identified from the qualitative descriptive thematic analysis. Finally, at the interpretation level, both datasets were merged to allow presentation of the results across dyads. Joint displays visually represent the insights gained from analyzing and bringing together quantitative and qualitative data (37). Pseudonyms were given to the participants for confidentiality and clarity of reading their narrative summaries.

## 3. Results

Three child-parent dyads completed the study (Marco, Ivan and Leo). Alma dropped out at week six of the intervention, and while the post-intervention assessment and interview were not completed, her results to that point are presented below. The start of the COVID-19 pandemic limited our ability to recruit one more participant to achieve a preferred sample of four children completing the intervention. Participants’ characteristics are described in Table 1. Supplementary Appendix C depicts each participants’ weekly playtime alongside reports of technical/daily routine challenges. Supplementary Appendix D showcases the total playtime per participant on each one of the BB mini-games. Individual experiences for each dyad are shared through narrative summaries linked to the study objectives.

**Table 1:** Participants’ characteristics.

Participant	Demographics	Participating parent	UL function	Experience with videogames
Marco	13 years old, left spastic hemiplegia.	Mother	Manual ability classification level (MACS) I (21)	Avid video gamer, mostly hand-held controllers.
			Grip strength (affected hand) 160.0 ± 20.0 mm Hg.	
			CHEQ (# of bimanual activities) 25/27.	
Alma	11 years old, right spastic hemiplegia, mild cognitive involvement	Mother	MACS Level II.	Some previous experience with computer games (played with the keyboard), Gameboy and Nintendo.
			Grip strength (affected hand) 126.7 ± 11.5 mm Hg.	
			CHEQ (# of bimanual activities) 22/27	
Ivan	10 years old, right hemiplegia, mild cognitive involvement, limited verbal communication.	Father	MACS Level I.	No previous experience.
			Grip strength (affected hand) 246.7 ± 20.8 mm Hg.	
			CHEQ (# of bimanual activities) 15/27	
Leo	8 years old, right spastic hemiplegia and Autism Spectrum Disorder.	Mother	MACS Level II.	Avid video gamer. Played Wii and/or Nintendo at home almost daily.
			Grip strength (affected hand) 36.7 ± 5.8 mm Hg.	
			CHEQ (# of bimanual activities) 4/27	

### 3.1. Marco

Marco was an avid video game player and owned a number of gaming systems and videogames.

#### 3.1.1. Participation in the BB intervention: motivational factors and aspects influencing achievement of the PTG

*“I liked the levelling up and unlocking new games the most”-Marco*

Marco identified a PTG of 15 min/day, 3 times a week, achieved in 10 of 12 weeks. Schoolwork was the main factor that impeded reaching the weekly PTG, but Marco usually made up for missed play time the following week as seen in the computer logs and described during the weekly phone calls.

*“Yeah, sometimes if I had, like, a long day at school, or if I had, like a lot of schoolwork to do, I would think that it would just be like a one extra thing to do (…) that I would want to kinda get it over quickly”. Marco*

On four occasions (as shared during the weekly calls), the videogame “*froze”* or did not load properly following which Marco simply restarted the computer and continued play. Marco’s mom (Marta) continuously encouraged Marco to meet his PTG. He used the multiplayer function in eight play sessions. Marta reported that “*he was more happy when someone would play with him”*. At the end of the 12-week program, Marco exceeded his total intervention PTG by 10%, achieving 596 min.

Marco’s most played games were Wizard’s Adventure, Bootle Band and Bubble Lab. He reported enjoying games with a high degree of challenge.—“*in Wizard’s Adventure, there were always new waves of new enemies coming at you, and it was fun, you know.”* Marta perceived that he also played the games that would get him the most points. Marta noticed he reached a plateau in the second part of the intervention. In response, at week 5 the research team (AK, DC) decided to switch him from time mode (default, mini-game finishes after 2 min) to life mode (mini-game finishes after you lose a set number of lives). This change was exciting to him because he felt more challenged describing it as “*good hard”*. Marco lost some interest towards the end of the intervention because he had unlocked and played all of the mini-games. At week 10, Marco said—“*I am getting very good at all the games; my scores are very high”*. His mother explained he was playing “*extra”* because he really wanted to reach level 100 before the end of the intervention. He made it to level 101.

#### 3.1.2. Clinical outcomes and usefulness of BB for UL therapy

*“it’s a work in progress [Marco’s UL motor improvements]”-Marta.*

Marco and his mother identified his COPM goals together (Table 2) and reported clinically important improvements in performance on 2 of the 3 goals post-intervention that were sustained at follow-up and attributed to the BB intervention. Changes in UL outcomes are presented in Table 3.

**Table 2:** Marco’s COPM scores across assessments.

Goal		Baseline	Post	Follow up
Tie shoelaces	Performance	2	**5** *	**7** *
	Satisfaction	1	**5** *	**8** *
Use left hand to access computer	Performance	5	**8** *	**8** *
	Satisfaction	8	**9**	9
Cut with knife and fork	Performance	6	7	7
	Satisfaction	6	**8** *	**7**

* MCID. Blue (bold) text represents positive change.

**Table 3:** Marco’s outcome measures results across assessments.

Outcome measures		Baseline	Post	Follow up
Grip (mm Hg)	Affected hand	160.0 ± 20.0	173.3* ± 15.3	xx
	Non-affected hand	[286.7 ± 11.5]	*[250.0 ± 10.0]*	
Range of motion (ROM)				
Shoulder flexion (degrees)		160	160	*150*
Shoulder abduction (degrees)		170	*150*	150
Elbow extension (degrees)		180	180	180
Wrist extension with fingers flexed (degrees)		60	75*	**70**
Wrist extension with fingers extended (degrees)		40	**60**	**60**
Wrist supination /pronation (degrees)		80	80	80
AHA* (0–100 points)		69	74*	74*
BBT* (blocks)		37	40*	42*
CHEQ (0–100 points)				
How do you think the child’s hand works?		54	**58**	**63**
How much time does your child need to do the whole task, compared to peers?		50	**53**	**64**
Is your child bothered by his reduced hand/arm function during this activity?		55	**57**	**64**
Number of bimanual activities		25	27*	27*

* MCID or SDC positive change. Blue (bold) and red (italic) text represent positive and negative change respectively. xx represents missing data due to equipment malfunction.

Marta thought the intervention was “*very good for him* [Marco]*”* because he constantly needed to work with his affected arm. She saw some improvements in Marco’s strength and confidence using his affected side, a decrease in asking for help to complete tasks, and being less bothered by things he had difficulty doing. He was also able to finish more tasks on his own. Marta told a story about a time Marco was able to perform an activity of daily living independently that he would usually ask for help on– “ *…I (saw?) … the confidence to do it* [i.e., using his affected hand to complete the activity of daily living in a social setting] *and I think it’s the most important thing, yeah”*.

#### 3.1.3. BB program as home-based therapy and recommendations for improvement

*“it’s more comfortable [therapy with BB], and if you need something you can just go and get it, right?”-Marco.*

Marco reported a preference for this type of therapy which did not involve travel; however, he recognized that not having an in-person therapist to answer questions could be a limitation. Marta found it beneficial that Marco could participate in the intervention on his own time while in his comfort zone (i.e., home). Recommendations for improvement offered by Marco and Marta were: (1) a better explanation of how to play some of the games.

*“Maybe he [Botley] could just explain some more, ‘cause… for his explanation of the games, some of them don't really, like, hit or something, like, hit home and I don't understand. Like for the baton thing I didn't really get how to do it until the people came home and I asked them about it and then they told me”- Marco.*

(2) adding more achievements to the game (e.g., if you buy more gear, you get more points or a new mini-game), and (3) having more mixed-reality games. Marta thought the game was “*childish”* for Marco’s age, and “*it wasn’t that much attractive”* compared to the other videogames he usually plays. Marta thought it would benefit the intervention to have added gaming challenges to BB.

### 3.2. Alma

Alma participated with her mother (Ana). Alman had some experience with videogames. Ana was hopeful that the gaming intervention might be motivating for her daughter.

#### 3.2.1. Participation in the BB program: motivational factors and aspects influencing achievement of the PTG

*“I'm good at the games [once she understood how to play them], but sometimes I don’t know how to play them”—Alma.*

Initially Alma was excited to play BB. Alma and Ana decided on a PTG of 20 min, 4 times a week. After week 1, the PTG was decreased to 3 times weekly, as Alma and her mother found that 4 times a week was onerous due to schoolwork and other responsibilities. Alma played on weeks 1 (once, 24 min), 2 (twice, total of 33 min) and 6 (once, 4 min). She did not play during weeks 3 and 4. Alma reported the videogame was “*a bit too childish”* and sometimes it was difficult to understand how to play some of the mini-games. She mentioned some games were “*boring”*, and others “*hard”*. However, Alma reported that she usually could figure them out after playing for a while. The monitoring therapist explained over the phone how to play the one game Alma reported struggling with, and referred Alma and her mother to the section of the user manual where they could find more detailed information with pictures. There were no reported technical barriers.

Ana reported trying to encourage her daughter to play because she thought BB was good for her. Alma said she enjoyed the multi-player option, but the play logs indicated limited use of this feature. During the home visit at week 6, Alma expressed that she did not want to continue with the study because of lack of time and loss of interest in the videogame.

#### 3.2.2. Clinical outcomes and usefulness of BB for UL therapy

Alma and her mother decided the COPM goals together (Table 4). Alma did not complete the post-intervention assessments or interviews. Baseline assessments scores are presented in Table 5.

**Table 4:** Alma’s COPM scores at baseline.

Goal	Performance	Satisfaction
Hold/carry items in right hand/arm at school	5	5
Carrying objects (i.e., grocery bags) with both hands at same time	3	5
Put hair in loose ponytail	1	2

**Table 5:** Alma’s scores at baseline.

Outcome measures		Baseline
Grip (mm Hg)	Affected hand	126.7 ± 11.5
	Non –affected hand	[410.0 ± 45.8]
Range of motion (ROM)		
Shoulder flexion (degrees)		145
Shoulder abduction (degrees)		155
Elbow extension (degrees)		150
Wrist extension with fingers flexed (degrees)		45
Wrist extension with fingers extended (degrees)		25
Wrist supination /pronation (degrees)		10
AHA (0–100 points)		57
BBT (blocks)		21
CHEQ (0–100 points)		
How do you think the child’s hand works?		57
How much time does your child need to do the whole task, compared to peers?		50
Is your child bothered by his reduced hand/arm function during this activity?		68
Number of bimanual activities		22

### 3.3. Ivan

Ivan participated with his father (Iker). His parents moved the furniture in his room around so Ivan could have enough space to play BB. Weekly phone calls were done only with Iker since Ivan had limited verbal communication.

#### 3.3.1. Participation in the BB intervention: motivational factors and aspects influencing achievement of the PTG

*“He had lots of time to play and the game was also very easy to follow”-Iker.*

Ivan and Iker identified a PTG of 20 min a day, 3 times per week. Whenever Ivan had a busy week, he tried his best to make up for the lost playtime later. He achieved his weekly PTG in 8/12 weeks. Overall, he played 945 min over the 12 weeks, an extra 31% above his total intervention PTG. The timing of the intervention was considered the “*perfect time”* by Ivan’s family, as it was summer vacation. Sometimes, Ivan would want to do preferred recreational activities instead of playing BB, but with some encouragement from his parents he would get started after which “*he would really enjoy it”*. The weekly call from the monitoring therapist also helped Ivan engage with BB, since as Iker said: “*It’s coming* [the encouragement to play] *from another person also* [not only from his parents]*”*.

Iker indicated Ivan’s favorite games were Color fill, Jetpack Bootle and Wizard’s Adventure. Ivan reached level 112 by the end of week 12. Ivan had fun and grew in confidence playing the game as the intervention progressed. He learned how to play by himself and “*that was something that I* [Iker] *was very happy to see him doing”*. Having a sense of ownership and control over the game “*the power to say yes or no* [when and with who to play]*”* was motivating and increased his confidence in playing. Iker described BB as “*fun to play”* for Ivan and his family, who often play together (12 multiplayer play sessions). There were no reports of technical difficulties during the intervention.

#### 3.3.2. Clinical outcomes and usefulness of BB for UL therapy

*“He (Ivan) is more able to use the right hand [on his own]” -Iker.*

Ivan’s COPM goals were identified by his father (Table 6). Changes in UL outcomes are detailed in Table 7. Iker perceived that BB allowed for a lot of movement repetitions, requiring Ivan to use his right hand to play. “*I'm surprised (…) this can do a lot of movements”*. Iker noticed several improvements on how Ivan used his hemiplegic hand: more spontaneous use and confidence in his ADLs (especially when using his toothbrush) and during videogame play, and a small improvement in the use of his affected hand’s fingers.

**Table 6:** Ivan’s COPM scores across assessments.

Goal		Baseline	Post	Follow up
Bimanually placing toothpaste on toothbrush	Performance	3	**7** *	**7** *
	Satisfaction	4	**10** *	**10** *
Opening fruit cup container using right hand to stabilize	Performance	3	**5** *	**6** *
	Satisfaction	4	**8** *	**7** *

* MCID. Blue (bold) text represents positive change.

**Table 7:** Ivan’s outcome measure results across assessments.

Outcome measures		Baseline	Post	Follow up
Grip (mm Hg)	Affected hand	246.7 ± 20.8	*233.3 ± 23.1*	233.3 ± 5.8
	Non-affected hand	[236.7 ± 30.6]	[243.3 ± 15.3]	**[256.7 ± 5.8]** *
Range of motion (ROM)				
Shoulder flexion (degrees)		160	**165**	160
Shoulder abduction (degrees)		170	*160*	*160*
Elbow extension (degrees)		180	180	180
Wrist extension with fingers flexed (degrees)		70	70	70
Wrist extension with fingers extended (degrees)		50	**55**	**55**
Wrist supination /pronation (degrees)		90	90	90
AHA* (0–100 points)		87	*84*	*84*
BBT* (blocks)		26	**34** *	**40** *
CHEQ (0–100 points)				
How do you think the child’s hand works?		44	**49**	xx
How much time does your child need to do the whole task, compared to peers?		37	**48**	xx
Is your child bothered by his reduced hand/arm function during this activity?		69	**70**	xx
Number of bimanual activities		11	**15** *	xx

* MCID or SDC positive change. Blue (bold) and red (italic) text represent positive and negative change respectively. *xx* represents missing data (at the time of follow up, time constrains prevented Iker to complete the CHEQ).

#### 3.3.3. BB program as home-based therapy and recommendations for improvement

*“it’s very safe, very easy, no money… no waiting”—Iker.*

At the beginning of the study Iker was “*nervous”* about using the videogame since he was concerned he would not be able to solve possible technical difficulties, or help his child understand how to play BB. They found “*the flexibility and the simplicity of the game”* very helpful to support Ivan’s play with BB. They did not need additional support from the research team to use BB. However, they indicated that it was reassuring to know that they could reach out for assistance if needed. For Ivan and his family, this was their first experience with videogames, and they “*loved”* the game; “*for him is playing but for us* [parents] *is therapy”*. Iker felt “*very strongly* [positive]*”* about this modality of therapy. Ivan had been waiting for UL therapy for several years. Iker “*never thought”* this would be a way of providing therapy for his son, but he found it very helpful (e.g., no waiting time, integrated within the family schedule, easy to use) and would recommend it to other parents because “*you get the therapy while you’re playing”*. Removing travel barriers and the low-risk intervention were very important to Iker. The family lived far from the hospital and taking Ivan to in-person therapy necessitated time off work. Recommendations for BB included the addition of more content to the videogame (e.g., more levels and mini-games)– “*at some point he* [Ivan] *finished playing with all the different things* [mini-games]*”*. Yet, Iker reported that reaching Level 100 did not hamper Ivan’s motivation to keep playing.

### 3.4. Leo

Leo played hand-held controlled videogames almost every day. He used his non-affected arm to push the buttons / move a joystick, and his right arm to hold the controller against his body. Leo’s mother, Lory, participated with Leo in the research study. Weekly phone calls were done mostly with Lory. Leo had an additional diagnosis of autism spectrum disorder. Both Leo and his mother were interviewed.

#### 3.4.1. Participation in the BB intervention: motivational factors and aspects influencing achievement of the PTG

*“[Leo talks about his busy weekly schedule]…So what I would do is … first find an actual time, to, like, play”—Leo.*

Leo’s PTG was 15 min/day, 4 times a week. This goal was achieved in 6 of 12 weeks. Leo had some weeks where it was hard to find the time to play (sickness, school, extracurricular activities), especially when one of his caregivers was away for work, however he still ended up achieving 701 min of active play time out of his 720 min total intervention PTG. Leo incorporated playing BB in his morning routine, usually before going to school. If he did not manage to play in the morning it was difficult to fit it in the rest of his day given his many extracurricular activities.

*“Yeah, so… 'cause we had it through for the 15-minute sessions a week. And that was generally reasonable ‘cause the only issue really where we had a whole bunch of things to do in the evenings, (…) there was a few times where, like, ‘oh’ we have something to do every night this week, how do we switch that [playing BB] in?” Lory*

Two technical difficulties were reported at weeks 2 and 4 (mixed reality object not detected). The research team tried to troubleshoot this by phone. At week 4 the problem persisted and a home visit was scheduled to fix it (AK). This caused some frustration for Leo according to his mother, because he had to wait to play the mini-game.

*“There was one game that wasn't working very well until it got recalibrated. The music one, yeah, … and there was a soccer ball one, with the batons. And it, you know, he got cranky with me, [because] you have to wait to play that one.” Lory*

Leo’s favourite games were Wizard’s Adventure (which Lory referred to as “*quite the work out”*), Bootle Kart, Astro Bootle, Jetpack Bootle, and Paint Baller. Leo needed some encouragement and supervision from his parents to play the game purposefully, since in the first few weeks he liked to “*intentionally bump into things* [the obstacles] *to get that big reaction”*, according to Lory. Leo was highly motivated by his chances of unlocking new games each week, winning against the computer, leveling up and collecting in-game rewards (i.e., game features). He needed some reminders to engage in playing BB, since he had other commercial videogames to play with. Nevertheless, Lory reported that most of the time it was Leo who took the initiative to play. He used the multiplayer option for 13 play sessions with his family, but also enjoyed playing against the computer and “*beating”* the Bootle. Lory mentioned that playing together improved his attention span towards the game. Overall, Leo was very proud of reaching level 92 because he “*worked really hard to get there.”.*

#### 3.4.2. Clinical outcomes and usefulness of BB for UL therapy

*“I don't know how much, you know, extra function he may or may not have gotten out of it, but I think it was very useful for reminding that we needed to use his arm (…) because he neglects it” Lory.*

Lory identified the two COPM goals (Table 8). Changes in UL outcome measures are shown in Table 9. Lory thought BB was mostly useful for reminding him to use his arm. She was unsure if it had helped him improve in his ADLs but noticed some “*progression”* with zipping up a jacket. Throughout Leo’s interview, there was an overall perceived value of usefulness. He felt playing BB helped him use his hand better, which motivated him to play.

**Table 8:** Leo’s COPM scores across assessments.

Goal		Baseline	Post	Follow up
Once zipper is engaged by caregiver, use right arm to stabilize against body to be able to pull up zipper	Satisfaction	2	**5** *	**6** *
	Performance	3	**6** *	**6** *
Push right arm through sleeve of loose-fitting shirt	Satisfaction	2	**8** *	**8** *
	Performance	2	**8** *	**8** *

* MCID. Blue (bold) text represents positive change.

**Table 9:** Leo’s outcome measure results across assessments.

Outcome measures		Baseline	Post	Follow up
Grip (mm Hg)	Affected hand	36.7 ± 5.8	36.7 ± 5.8	36.7 ± 5.8
	Non-affected hand	[220.0 ± 20.0]	*[206.7 ± 11.5]*	**[236.7 ± 15.3]** *
Range of motion (ROM)				
Shoulder flexion (degrees)		135	**140**	**155**
Shoulder abduction (degrees)		150	**170**	**175**
Elbow extension (degrees)		170	170	170
Wrist extension with fingers flexed (degrees)		0	0	0
Wrist extension with fingers extended (degrees)		0	0	0
Wrist supination /pronation (degrees)		0	0	0
AHA* (0–100 points)		27	27	**32** *
BBT* (blocks)		31	**36** *	**40** *
CHEQ (0–100 points)				
How do you think the child’s hand works?		22	*17*	*14*
How much time does your child need to do the whole task, compared to peers?		46	*42*	*38*
Is your child bothered by his reduced hand/arm function during this activity?		58	*44*	*47*
Number of bimanual activities		4	*1*	*0*

* MCID or SDC positive change. Blue (bold) and red (italic) text represent positive and negative change respectively.

#### 3.4.3. BB program as home-based therapy and recommendations for improvement

*“Yeah. I think one of the things was ‘cause you know, you're often told oh do exercises at home, so do these things, but they're not fun. So the kids are I don't want to do them, and you're sitting there [unclear] then I don't want to make you do them… but the game actually it’s like hey let’s go do this fun thing” Lory*

Leo enjoyed this type of therapy, especially the fact that he did not need to travel. Leo was also aware of the time expense of in-person therapy experienced by his mother “*and everyday I would have to come here, my mother would too”*. For Lory, this type of therapy was a way to make home-therapy fun, and to decrease the pressure of the parent having to be the “*home therapist”*. Lory mentioned that she and her husband would usually try to be present while Leo was playing BB as their “*together activity”* but sometimes “*it was like oh, you need to do that while I’m making dinner”* which appeals to the busy family dynamic with two young children. Recommendations for improving the system related to adding more competitive and action-driven mini-games that could be played with a more diverse movement repertoire.

*“Maybe you need more games where you can blow stuff up. But, yeah, ‘cause he does like that crash boom bang, you know. More action ‘cause, (…) he was always crashing into the things to get the… the reaction.” Lory*

### 3.5. Interview themes

Four themes relating to the participants’ overall experiences with BB as a tool for UL home-based rehabilitation were identified across five interviews with children (Marco, Leo) and parents (Marta, Iker, and Lory), and are described as follows:

1) Intrinsic motivators fostered play engagement:

While each child found their own meaning in the game and had different preferences with respect to specific mini-games, what made Bootle Blast “*fun*” overall was its capacity to inspire feelings of autonomy, mastery and relatedness.

*“It was fun for the most part, I liked it [BB].”—Marco*

a) *Autonomy:* The ability to navigate the system independently and make choices within the game and intervention (e.g., multiplayer option, 13 mini-games to choose from, when and for how long to play) was valued by children and their parents, and increased children’s sense of ownership towards the use of BB during the intervention. Children’s confidence to face in-game challenges increased as they acquired gaming experience and mastery of the system.*“He has a [the] control, right? So he’s the… he’s the owner of the game. So he enjoys it…So he had the power to say yes or no.. So that helped him…To get more… more confident.”- Iker*
b) *Mastery:* Children expressed great pride in their BB accomplishments (achievements, unlocking games, movement), all of which were intrinsically motivating.*“…and he said, “yes I did it!” [reached level 100], and it was good.”- Marta*

Conversely, running out of game content and achievements (particularly towards the end of the intervention) negatively impacted motivation to play. All families suggested that additional game content (more mini-games to unlock, more collectables, more movement variety) would enhance the game and help to sustain interest beyond the 12 weeks.

*“Maybe towards the end. When I, like, already unlocked every game, there was, like, you know, I played every game, I kinda got bored after a while… so I mostly played just the few, like, the three or four games that interest me the most…near the end.” Marco*

Other deterrents to motivation occurred when children experienced technical difficulties or found BB tasks difficult to learn. This was observed in the case of Alma and also reported as an area for improvement by Marco who suggested that BB’s main character should explain more specifically how to play each mini-game to make it easier to learn.

c) Relatedness: allowed for other family members to engage with the intervention making it “*fun”*. Children enjoyed playing with their siblings or parents but winning against the “*computer”* also fostered a sense of connectedness, as mentioned by Lory and Leo.

*“What helped me a lot is if I was playing with another person.”—Marco*

2) Virtual play for real-world therapy gains:

Parents perceived that higher use of the hemiplegic UL in BB increased children’s confidence to use their hemiplegic hand in their everyday life. Parents reported seeing more spontaneous use of the hemiplegic UL and a reduced need to remind their children to incorporate their hand during bimanual activities.

*“… the expectation was, like, having the left [hemiplegic] side be more useful and he’s been more confident. And I think it [BB] definitely contributed to that… he’s able to do things now without, like, asking for help, or without, like, … being sensitive to do it and not be able to finish the task, I think it definitely helped”—Marta.*

*“It [BB] did remind him he should be using his right arm, which was good…’cause, yeah, he often forgets, that he has it”—Lory.*

Parents and children also gave specific examples of how they perceived their abilities had improved as a result of the BB intervention, often with reference to their COPM goals. While for Marco and Leo, BB was most valued for the “*fun”* it could provide rather than its therapeutic value, both felt that overall BB helped them to improve in the way they used their affected hand.

*“[using my] … left-hand [in] the computer, you know, typing more. That [BB] definitely helped [with]”.—Marco.*

*“Watch this [to interviewer]. I can even open the door. That’s a new thing that I learned how to do [because of BB]”—Leo*

3) Therapy on demand:

BB was valued for its flexibility and convenience which facilitated its integration into the families’ varying routines and dynamics. While Marco’s weekly routine fluctuated from week to week due to school commitments, Ivan did the intervention during summer break, and Leo mostly played at a fixed time each morning because it was what worked better for him. BB allowed children the flexibility to adapt their play time as needed and to participate in the intervention in the convenience and comfort of home which made achieving the PTGs manageable for the families. As an intervention, BB was able to bridge some of the accessibility barriers associated with therapy (e.g., time and travel constraints for Marco and Leo, and waiting list for Ivan).

*“He [Marco] was in his comfort zone, like, in his own place” Marta.*

*“This is very good, I would really recommend if you have this options, give it to the kids because it’s very well set up…… you get the therapy while you’re playing. So it’s double thing that you get…So I really love that, I like the approach, it’s very cost effective I think, you don't have to wait for the waiting list. That’s very bad [the waiting], you don't need to travel, it’s in the home”.—Iker.*

*“…coming to the hospital [for therapy], every single day…that would take a time. And…I only got time from 4:30 to 8 o’clock (…) And everyday I would have to come here [the hospital], my mother would too (…) [I prefer] playing that [BB] on the computer instead of coming to the hospital”—Leo.*

4) Blame it on the game! Shifting the onus from the parent to the game.

While BB is in a different category than preferred activities like entertainment gaming or watching TV, a gentle reminder from parents was usually sufficient to get children playing and once the activity was initiated, it was usually enjoyed. The ability for children to do the program relatively independently facilitated its adoption into the busy home life. At the same time, BB also helped to relieve some of the parental stress associated with managing home-based therapy programs.

*“Because I cannot be there all the time to tell him that okay use the right hand… Right? He even get upset on me like why are you telling me all the time? But with this one he has to because there is… there is no person telling. It’s the system…”—Iker*

### 3.6. Integration of phases I and II

This mixed methods case series describes children’s home experience with a new movement tracking, mixed reality therapy game, BB. Quantitative and qualitative results were merged to better understand adherence to the weekly PTG (objective 1), changes in hand/arm clinical outcomes (objective 2), and families’ experiences with the intervention (objective 3). Tables 10, 11 show joint displays describing key findings and meta-inferences (integrated views of findings from both quantitative and qualitative strands) (38) for objectives 1 and 2 respectively.

**Table 10:** Results summary and joint display for objective 1: adherence to PTG.

Participant	Quantitative	Qualitative	Meta-Inferences
Marco	PTG was 15 min / 3 days per week (540 min in 12 weeks). Played 596 min. Most frequently played mini games were those with the highest degree of challenge and that tended to award the most points to the player. Multiplayer option was used in 10 out of 40 days of play.	Did not play in busy weeks but compensated for the lost time in following weeks.	**Convergence:** Playtime varied from week to week due to extrinsic factors, but if the individual is intrinsically motivated by BB (e.g., find meaning in unlocking new mini-games, achieving high scores and attaining all the collectables), they may strive to make time in other weeks in order to achieve their PTGs.
		Leveling up and feeling challenged were important motivational factors to play.	
Alma	PTG was 20 min/3 days per week. Played a total of 61 min. Multiplayer option used 1 time for 4 min. Logs showed Alma played less than 10 min each time she turned on BB, with no play time in weeks 3 and 4. Alma dropped out at week 6.	Alma reported enjoying playing with her family and using BB in each week of the intervention.	**Divergence:** Characteristics of the family and the individual influence play motivation (lack of time, physical / cognitive challenges, perception of age appropriateness, presence of play partners). Disagreement between system logs and participant’s may be due to reporting bias or misperceptions. This suggests the importance of system-tracked play metrics for monitoring interventions.
		Reported that the game was too childish, and some mini games were difficult to play.	
		Monitoring therapist perceived physical and cognitive challenges to play caused frustration.	
		Reason for drop out was loss of interest and no time to play (she had family responsibilities and schoolwork).	
Ivan	PTG was 20 min/3 days per week (720 min in 12 weeks). Played 945 min. Ivan’s play sessions tended to be longer than his PTG, although he played less days per week.	Timing was beneficial for adherence as it was summer break.	**Convergence:** Family support, multiplayer option, having the system during vacation time, and the perceived BB ease of use were crucial in sustaining adherence to the intervention.
		Parent encouragement, weekly phone calls and playing with someone else were key motivators.	
		Ivan’s sense of ownership and increase in confidence while using BB also facilitated his motivation to play.	
	Some of his most frequently played mini games included a multi-player option, which was used in 12 sessions during 20 days of play.		
Leo	PTG was 15 min/4 days per week (720 min in 12 weeks). Played 701 min.	Play time was difficult to fit in during weeks with additional schoolwork or activities. For Leo and his family, it worked well to play BB as part of his daily routine at a specific time in the morning. Parental involvement (including playing the game), in-game rewards and a sense of mastery (reaching level 92) encouraged Leo to play BB. Having other videogame consoles was a competing interest that deterred BB play.	**Convergence:** Lack of time, either due to a busy family schedule or life events, and competing interests can be barriers to playtime adherence, however setting up a routine and parental support, particularly as play partners, are important and motivating supports.
	Most frequently played games included a multiplayer option (player 2 or against the computer). Over 37 days of play Leo played 13 times with someone else.		

**Table 11:** Results summary and joint display for objective 2: changes in UL outcomes.

Participant	Quantitative	Qualitative	Meta-inferences
Marco	*Improved post BB:* grip strength, wrist extension, AHA, BBT, CHEQ and COPM.	Marco thought the intervention helped him improve the use of his affected UL in some of his ADLs.	**Convergence:** Improvement in most clinical outcomes gave a sense of usefulness of the intervention from both child and mother perspectives, specifically in relationship to the COPM, where Marta gave examples about how these improvements “looked” in Marco’s ADL’s. of note, shoulder ROM is the only outcome that did not improve. However, reference to shoulder movement did not arise in the qualitative data.
	*Decreased post BB:* ROM for shoulder ABD.		
		Marta thought playing BB gave Marco more confidence in using his affected UL, especially in cutting with a knife and fork.	
Ivan	*Improved post BB:* shoulder flexion, wrist extension, BBT, CHEQ, and COPM.	Ivan’s father reported an overall improvement in Ivan’s use of his affected UL.	**Convergence:** Ivan’s father perceived his child using his affected UL more spontaneously and with more confidence in ADLs. He also gave specific examples about improvements related to toothbrush use and finger dexterity, supporting Ivan’s improvements in several clinical outcomes, especially the ones related to the COPM. Reference to a decrease in range of motion, grip strength or accuracy was not present in the qualitative data.
	*Decreased post BB:* grip strength and shoulder abduction.	Gains were especially related to more spontaneous use and increased confidence in using his affected hand and fingers.	
Leo	*Improved post BB*: shoulder flexion and abduction, AHA, BBT, and COPM.	Both Leo and his mom thought BB was mostly useful for encouraging use of the affected UL. Leo’s mom gave an example on how it helped with activities like zipping up a jacket but was not sure if the game had influenced the use of his affected hand/arm in other ADLs.	**Convergence:** Most of Leo’s improvements on clinical outcomes related to the spontaneous use of his affected hand which Leo and his mom both felt was encouraged by BB. The lack of increase in scores for the CHEQ was consistent with Leo’s mom hesitation on the extent to which BB had helped him improve in his ADLs. This perhaps suggests an increase in capacity for use of the hemiplegic had that has yet to transfer into performance in ADLs.
	*Decreased post BB:* CHEQ.		

## 4. Discussion

This study investigated the use of a novel mixed-reality and movement-tracking videogame for home-based rehabilitation for children with HCP. The intervention was novel in terms of technology used, as well as its family-centered approach, which empowered families to select their therapy and playtime goals. Overall, three of four parent-child dyads had positive experiences with BB, considered it useful for UL therapy and achieved their PTGs throughout the intervention. Improvements in clinical outcomes varied among children, with the BBT and COPM showing consistent positive change. One participant did not find BB appealing and struggled to fit it into her weekly routine. The case series design enabled the generation of rich insights and key learnings about this new intervention. Key learnings are discussed below for each research objective.

### 4.1. Engagement through the intervention (objective 1)

• Key components to facilitate engagement are a family-centered approach to intervention design, and a mix of intrinsic and extrinsic motivators.
• Finding the right family-child intervention fit is essential.

Family-centered interventions can align better with real world implementation (19, 39). In BB, a family-centered approach and self-directed PTG were designed to promote feelings of autonomy (control on what to play, when and for how long), competence (confidence that the intervention activities and level of challenge were manageable) and relatedness (connection through play with family) (40), all of which were important intrinsic motivators in the intervention. Parental reminders to initiate play was the key extrinsic motivator. Weekly calls from the monitoring therapist were perceived as engaging for Ivan and Leo, while not as helpful for Alma and Marco. However, these weekly communications played an important role in understanding how the intervention evolved for each participant and provided insights on how to adapt it to sustain the child’s engagement (e.g., when switching Marco from time to life mode to increase his challenge) (9). In contrast, technical issues, difficulty understanding how to play the game (lack of competence), characteristics of the home environment (e.g., number of caregivers involved), and competing interests (e.g., other gaming consoles) or responsibilities (e.g., schoolwork, extracurricular activities) were barriers to engagement, particularly if the child’s interest in the game was low. These findings align with previous studies (12, 39, 41). Family-identified improvements centered on providing more game content and opportunities for continued challenge. Better game tutorials and minimizing technical issues will also be essential to avoid user frustration and reduce the time the monitoring therapist needs to spend addressing these issues.

Interventions that fit the family routine, and that are perceived by participants as easy to implements and useful for therapy increase the overall feasibility and adherence to home-based UL programs (9). In this study, ensuring that the child and family-intervention fit was good was essential to promoting engagement. Having a goal rather than a fixed play schedule felt less onerous for participants and was sensitive to their routines, demonstrating that families could select and achieve a PTG that leads to positive changes in clinical outcomes with BB. In Alma’s case, the monitoring therapist perceived that Alma experienced frustration when she did not understand how to play the mini-games. Additionally, games that required more physical endurance were challenging for her. These barriers could be addressed through system improvements and more therapist involvement at the outset. However, other factors (lack of interest in the game, difficulty fitting into the daily routine) suggested that perhaps a different intervention (e.g., with a clinician present at a protected time) could have been a better fit for Alma’s family. More concentrated efforts to establish a child and family-intervention fit (e.g., via pre-intervention interviews, trial play sessions) could set families up for success both in future research trials and in clinical practice.

### 4.2. UL clinical outcomes (objective 2)

• Positive changes in hand use were observed in the game and in real life.
  • Impact on function in daily life skills was observed through consistent positive change in the COPM goals.
• Use of mixed methods was helpful to understand and interpret change in UL clinical outcomes.
• Future studies can benefit from measures that capture spontaneous hemiplegic hand use in ADLs and changes in competence related to hand use.

BB prompted use of the hemiplegic UL during game play while allowing children to take ownership (e.g., choosing which mini games to play and for how long) and develop mastery as they became experienced players. Participants valued this difference from traditional therapy programs where the therapist usually oversees the session. They also perceived those skills practiced in the game lead to an increase in the affected UL use and confidence during ADLs. Positive impact on performance and related satisfaction of ADLs was evidenced by consistent positive change on the COPM goals for all three participants who completed the intervention as well as on the CHEQ for two of three participants. Parents’ interviews and weekly reports were essential to better understand the changes in the children’s UL clinical outcomes, as caregivers were able to identify small daily functional improvements that were not detected by the clinical measures. While changes in clinical measures occurred where children had more room for improvement at baseline, some observational assessments showed decreased scores. However, there was no evidence from the interviews that families perceived an accompanying decrease in function. These conflicting findings could have occurred due to (1) decreased participant performance at the time of assessment (e.g., it was difficult to engage Leo in his follow up assessment), and (2) different constructs being assessed across the two data sets. Clinical assessments focused on measuring UL ROM, performance, strength, and dexterity, yet the most reported change in the interviews was improvement of UL awareness/neglect on the affected side. As often seen in traditional development, it may be that greater use of the affected UL (quantity) needs to happen before quality gains can be made (42).

Treatment intensity and goal-oriented interventions has been related to improvement in UL motor function in children with HCP (9, 41, 43). Recent literature has identified an average of 14–25hrs of practice to achieve meaningful changes in UL outcomes in the COPM (43). Even though the participants range of practice time with BB was below this average (10–12 h), positive MCIDs in the COPM were achieved. “Dosage” is usually only one component of a mix of factors influencing effectiveness of an intervention (9, 41, 43). As showcased in this study, and in line with existing literature, the engagement and the perceived value of the intervention are key factors to be considered, as well as outcomes focused on intervention enjoyment, and functionality and efficiency on bimanual activities (43–45).

It is important to stress that our results are not intended to be generalized to other gaming systems. Minutes of active play time with BB may be very different with respect to intensity and motor practice than the same time with a different gaming system. Rather, this study emphasizes the importance of prioritizing the family’s role in designing an intervention that can suit their lifestyle, the importance of considering characteristics of the technology and of the child’s play style when trying to understand the relationship between “dosage” and clinical outcomes. Future research with the BB intervention should investigate its impact on UL neglect, spontaneous use and competence in ADLs through more in-depth quantitative and qualitative measures.

### 4.3. Learnings on the family experience (objective 3)

• BB supported family choice and autonomy.
• BB as a tool for UL therapy was valued for different reasons by different families.

The convenience of accessing the intervention from home and on their own time was very valuable for children and parents. The overall sense of autonomy the families felt when using BB boosted engagement in the intervention and increased its perceived value as an option for UL motor therapy. Parents appreciated having the choice of how much involvement to have during play time as opposed to traditional home-based therapy, which is usually more directly supervised and hands on. Yet, parental support was still important to keep children engaged. While BB was not perceived as a leisure activity like entertainment gaming or watching TV, a gentle reminder from parents was sufficient to get children playing and once playing, they enjoyed the experience. BB also fostered social play, with all participants mentioning amusement in playing with family members.

Participating families had different backgrounds, needs, and routines. For the three parents who had positive experiences with BB, what they valued most about it differed. For Marco’s family, the value was how comfortable and “easy” it was to do the therapy at home. For Ivan’s family, BB was the solution to a frustrating long stretch on a waiting list to access UL therapy. For Leo’s family, it was the fun component of it and relieving the burden on the parent to serve as a “home-therapist”. These findings strengthen the idea that one size does not fit all, and the importance of using a family-centered approach when implementing home-based videogaming interventions to set the stage for success.

## 5. Limitations and future work

This study was affected by the COVID-19 pandemic, halting recruitment after our fourth dyad. While four participants are preferred in multi-case mixed methods studies, one of our case datasets (Alma) was incomplete as the family did not wish to participate in follow-up interviews or functional assessments at the time of dropping out of the study. Of note, after completion of the four participants’ use, enough information had been collected to inform significant system improvements which were carried out during COVID-19 lockdowns including: animated tutorials and instructions on how to play, more rigorous quality assurance testing to reduce technical issues, a system to enable remote access to BB data, and transitioning to a new hardware platform (Orbecc Persee—a plug-and-play 3D camera-computer) to replace the discontinued Kinect 2. A study of BB v2 using a single-case experimental design with 15 children with CP is underway, applying the family-centered approaches used in this case series including a phased recruitment process to better establish child and family-intervention fit. Additionally, we will also be more deeply exploring the role of the monitoring therapist in the BB intervention.

As only young males completed the study, the absence of female participants represents a limitation in our results which will be a focus of future research with purposeful recruitment. Extensive involvement of the primary author from study design to publication can create sources of bias, especially in relation to the qualitative data (e.g., confirmation, question-order or wording biases). This was mitigated by following principles of qualitative rigour such as having different data sources and methods; member checking, peer debriefing, and keeping an audit trail. Both interviewers had some experience interviewing children and pilot tested the interview guide with DC before their interaction with participants. However, neither was a trained qualitative researcher.

## 6. Contributions

This work provides a demonstration of an innovative videogame home intervention (BB) with a family-centered approach. Results demonstrate that BB can be successfully used at home to facilitate access to, and encourage engagement in UL rehabilitation care in children with CP. The use of mixed methods allowed a better understanding of which components of videogaming interventions can be successful, and feedback on which elements need improvement. The integration of data sets provided deeper insights on why clinical outcomes change and how engagement can be sustained in this type of interventions, while strengthening the results when concordance or divergence of quantitative and qualitative data occurred. This paper emphasizes the importance of child and family-centered therapy approaches, where the clients’ experiences are as valuable as the clinical measures. Making therapy “fun” and accessible via videogames can have a positive impact on clinical outcomes and on the value children with CP and their families give to different and novel rehabilitation approaches.

## Data Availability

The datasets presented in this article are not readily available because the participants of this study did not give written consent for their data to be shared publicly or to be used for secondary data analyses. For this reason, data is not available for sharing. Requests to access the datasets should be directed to ebiddiss@hollandbloorview.ca.
