# Supplementary material for: Understanding a videogame home intervention for children with hemiplegia: a mixed methods multi-case study
Source: Front Med Technol. 2023 Jul 12;5:1217797. doi: 10.3389/fmedt.2023.1217797 (PMC10368996; doi:10.3389/fmedt.2023.1217797)
Supplement: Supplementary file 1 [file Datasheet1.docx]

Supplementary Material

Understanding a video game home intervention for children with hemiplegia: a mixed methods multi-case study

Daniela Chan-Víquez, Ajmal Khan, Sarah Munce, Darcy Fehlings, F. Virginia Wright, Elaine Biddiss*

*** Correspondence:** Elaine Biddiss, PhD: ebiddiss@hollandbloorview.ca

# Table 12. Mini-games Summary (Appendix A)

| Mini-game | Game play | Targeted movements | Toys needed | Player options |
| --- | --- | --- | --- | --- |
| 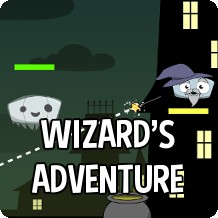 | Player defends a castle against a wave of ghosts. When you see a Ghost Bootle approaching, move your arm up to the side (i.e. shoulder abduction) to aim the Wizard’s wand. Hold your arm in position until the Ghost Bootle is zapped away. Extend your elbow for extra range. Rest your arm by your side to recharge your wand in the magic pot. | Shoulder abduction  Elbow extension  Plays with most affected upper limb | None | Single player  Multiplayer co- operative |
| 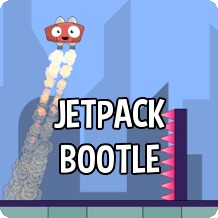 | Player moves targeted arm up and down in front of them (e.g. shoulder flexion) to avoid obstacles in this endless flyer game. | Shoulder flexion  Plays with most affected upper limb | None | Single player |
| 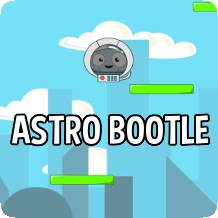 | Player leans left and right to jump from platform to platform, avoiding aliens and collecting power-ups in this endless jumper game. | Trunk lean | None | Single player |
| 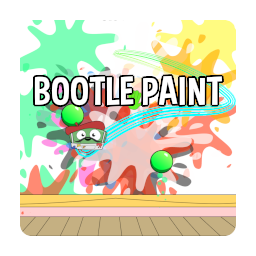 | Player reaches for moving coloured targets in order to splatter them on the painter’s canvas, and avoids “bombs”. Player must switch hands when prompted. | Shoulder abduction/flexion  Cross body reach  Elbow extension  Plays with both upper limbs | None | Single player |

**Appendix A (continued)**

| Mini-game | Game play | Targeted movements | Toys needed | Player options |
| --- | --- | --- | --- | --- |
| 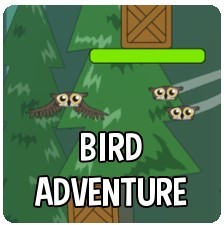 | Player explores an open-ended world using a “flapping” arm movement to travel as a Bootle Bird. There are many interactive elements to the map, and 9 special tasks to complete in order to collect all of the stars. Flapping with the left arm moves the bird right and vice versa. | Shoulder abduction  Bilateral coordination | None | Single player |
| 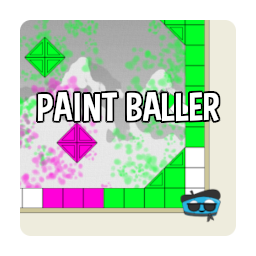 | Player performs a “clap” motion to fire paint balls and colour fill the most territory in this competitive game. To fire the paint ball, make sure you open your arms wide enough, and then clap! The on-screen hand icons will turn white when you are ready to fire. | Bilateral coordination; bringing hands to mid line. | None | Single player  Multiplayer competitive |
| 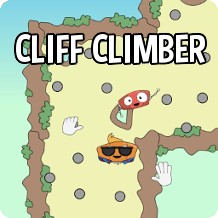 | Player reaches to grab handholds and climb a mountain, collecting Bootles as they go. You can move just one hand at a time while your other hand remains anchored to the last handhold position. Hover over a handhold to grasp it. Then reach for the next handhold with your other hand. | Bilateral coordination  Cross body reach  Elbow extension  Shoulder abduction/flexion | None | Single player  Multiplayer competitive |
| 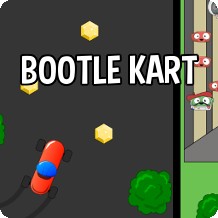 | Player steers an imaginary (or real) steering wheel and navigates their kart to avoid obstacles and collect gems. | Bilateral coordination | None | Single player  Multiplayer competitive |
| 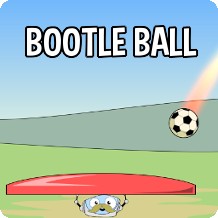 | Player rotates a baton (wrist supination) to control the position of a platform and keep a bouncing ball in the air. | Wrist supination and pronation  Plays with most affected upper limb | Red-green baton | Single player  Multiplayer competitive |

**Appendix A (continued)**

| Mini-game | | Game play | | Targeted movements | | Toys needed | | Player options |
| --- | --- | --- | --- | --- | --- | --- | --- | --- |
| 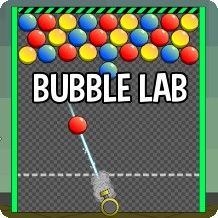 | | Player picks up and shows the camera a Mega Blok of the colour they want to use. Hold the Mega Blok up in front of you above your shoulder. Make sure that the camera can see the block clearly. Try to connect 3 bubbles of similar colour to pop them. | | Grasp and release | | Red, blue, yellow Mega Bloks | | Single player |
| 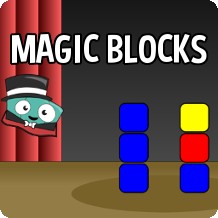 | | Player replicates on-screen coloured towers using real- life Mega Bloks. Once you have built the tower, hold it up in front of you above your shoulder in order for the game to score your tower. Make sure that the camera can see the block clearly. | | Grasp, manipulation and release | | Red, blue, yellow, green Mega Bloks | | Single player  Multiplayer competitive |
| 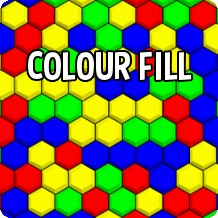 | | Player picks up and shows the camera a Mega Blok of the colour they want to play. The object of the game is to colour fill the grid in as few moves as possible. Hold the block up high in front of you and make sure that the camera can see the block clearly. | | Grasp and release | | Red, blue, yellow, green Mega Bloks | | Single player  Multiplayer competitive |
| 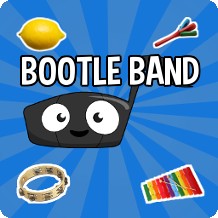 | Player manipulates real-life instruments to create a Bootle Band. Game tasks involve “busking” to collect coins (level 2); recording songs (level 3); and playing in a concert (level 4). | | Grasp, manipulation, and release | | Tambourine Maraca Castanet Xylophone for levels 2+ | | Single player  Multiplayer collaborative | |

# Interview Guide (Appendix B)

## Post-study interview – Child

1. Can you tell me about being involved in the Bootle Blast program?
   1. Overall experiences during the entire intervention / How did it feel?
2. What did you like best about the Bootle Blast program?
   1. Were there any games the child liked best? / Things the child liked/disliked at the beginning, middle and end of the intervention. General idea on how their “feelings” towards the game changed over time.
   2. Did the child think it was fun?
3. What did you not enjoy about the Bootle Blast program?
   1. Same probes as question 2
4. Can you tell me about anything that made it hard for you to do / accomplish your play time goal for the Bootle Blast program?
   1. Feelings, time constraints, school projects, time during the holiday etc.
5. Can you tell me about anything that helped you to keep going with the Bootle Blast program?
   1. Feelings / experiences about multiplayer mode / parents’ engagement and/or encouragement / any other possible motivational factors.
   2. Was it better when the child played with someone else? Did mom/dad/sibling encouraged child to play? Did the child do it because it could help them get better at using the hemiplegic arm?
6. Have you noticed any differences in the way you use your affected arm in daily activities?
   1. Does child feel playing the game has helped them?
7. How did you find doing your therapy on a computer at home compared to going into the hospital for therapy?
   1. Is it better? Worse? As good? Why... which one does the child prefer and why?
   2. Feelings about both types of therapy.
8. If you could change something about the Bootle Blast program, what would it be?
   1. What would you have done differently?
   2. Do you have any ideas on how we can improve the game?
9. Is there anything else you would like to tell me?

## Post-study interview – Parent

Can you tell me about how you and your child experienced your participation in the Bootle Blast program?

Overall experiences during the intervention.

What were the best things about your involvement?

Was it useful for doing therapy? Did they like it? Was it fun? did it help with the therapy goals?

Was it feasible to achieve the play time goal?

What were the most difficult things about your involvement?

Was it hard to find the time to play, did it become boring with time, did parents see the purpose on playing Bootle Blast?

What were your expectations of being involved in the Bootle Blast program?

Were their expectations satisfied? If not, why?

Do you think Bootle Blast impacted on your child’s ability to participate in their daily life tasks?

Did the parent see change in the goals set on the first day? Did playing Bootle Blast help the child work towards those goals? Why?

How did you find this mode of therapy (home based) compared to previous types of therapy your child may have received?

What things would you change about the Bootle Blast intervention?

What can be improved, removed, or done completely different?

What things did the parent consider were good, useful or/and valuable about the intervention for them and their child?

# Figure 2. Total Bootle Blast playtime per participant, week, and timepoints at which issues (technical or other) were reported (Appendix C).


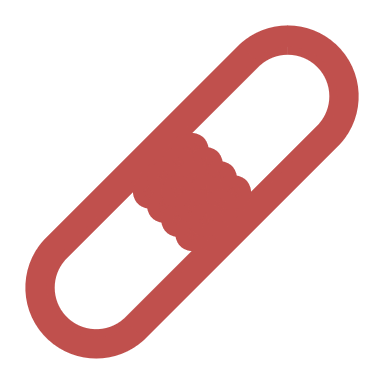


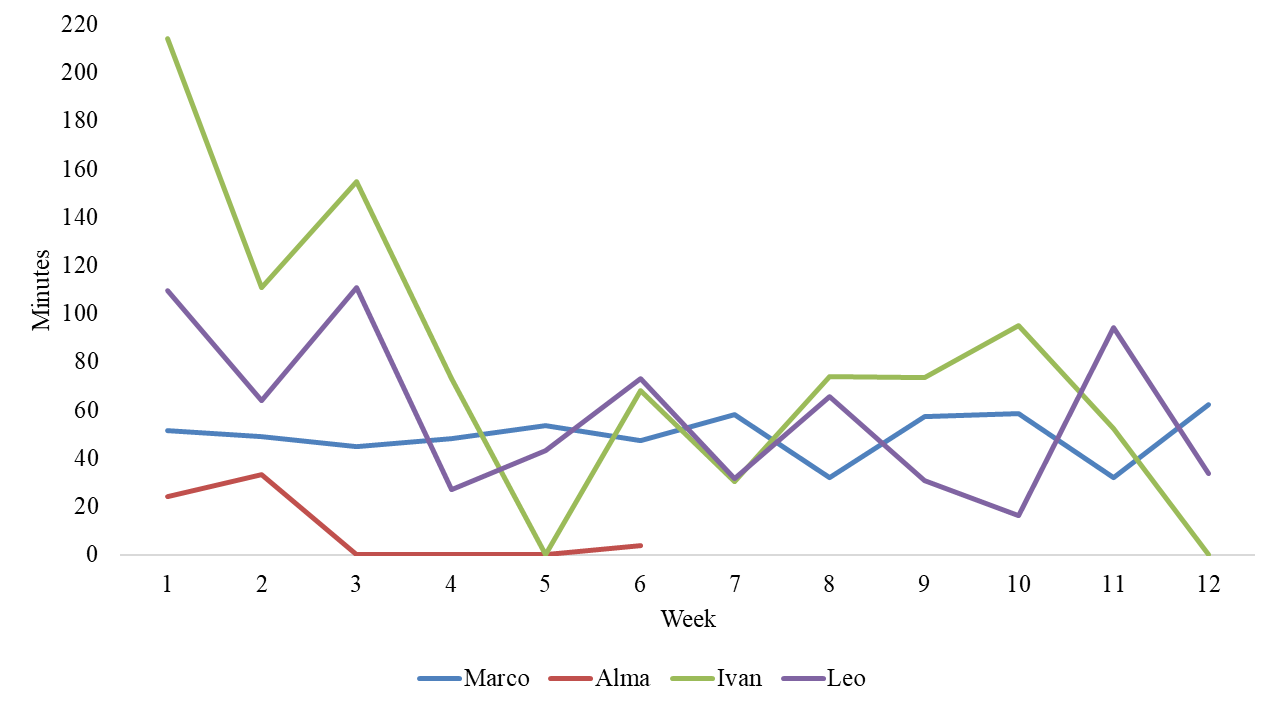

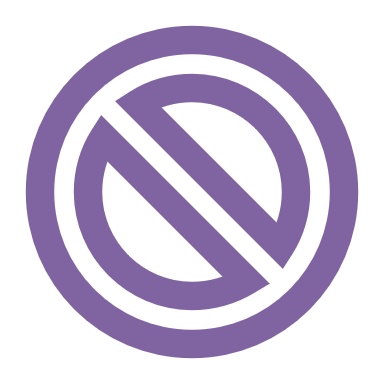

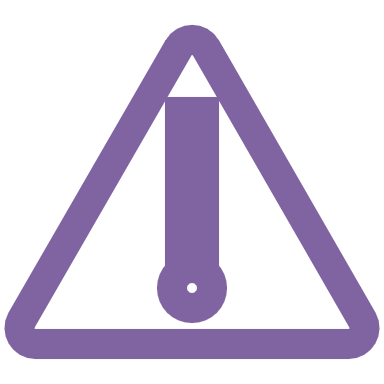

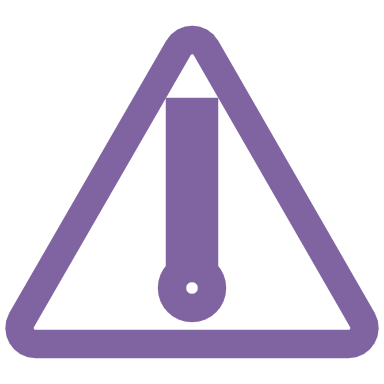

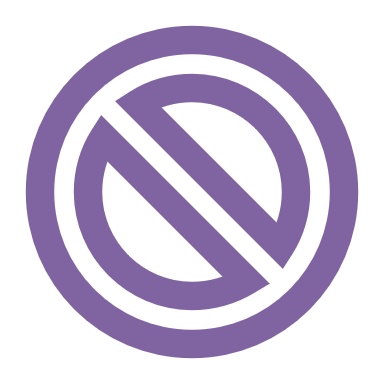

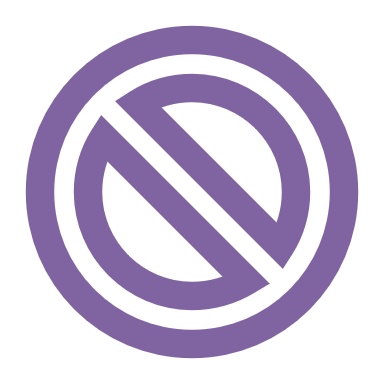

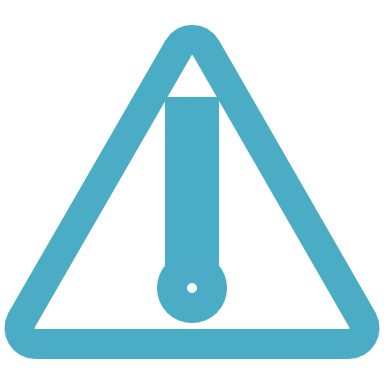

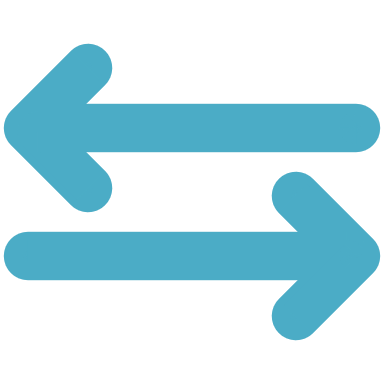

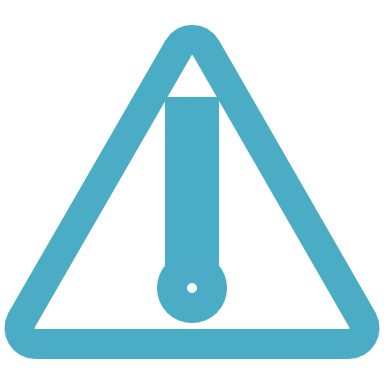

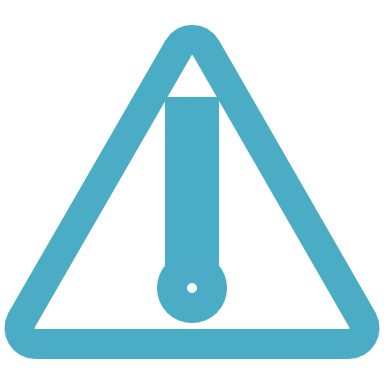

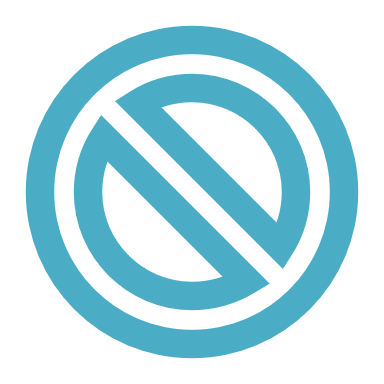

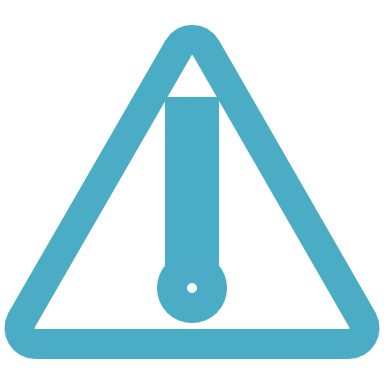

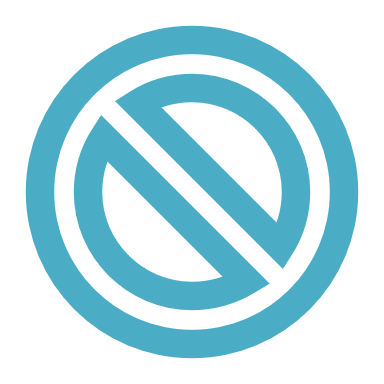

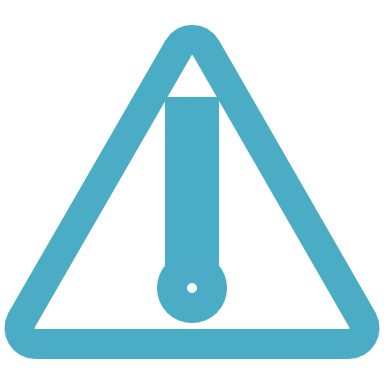

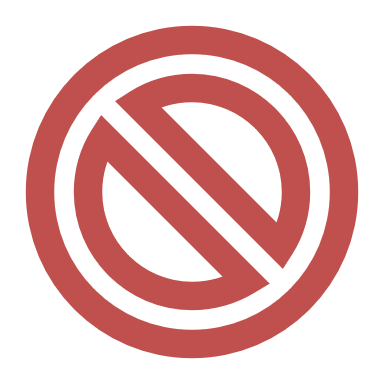

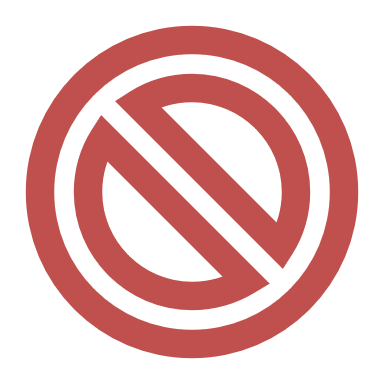

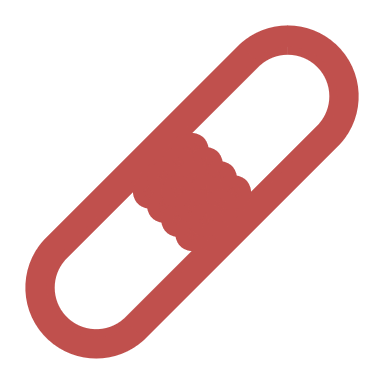

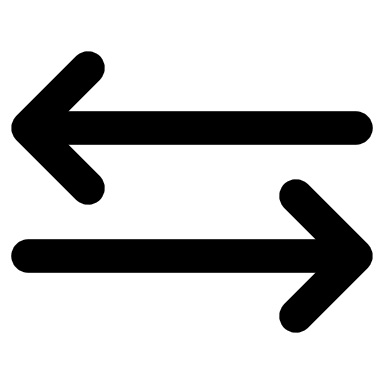

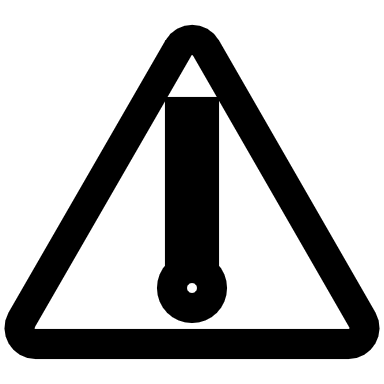

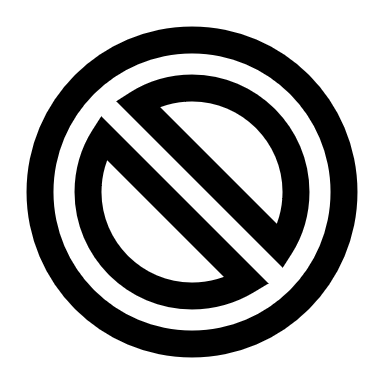

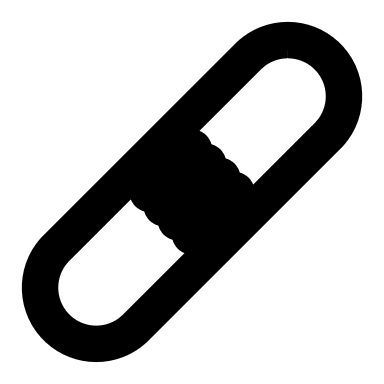


Switched from *time mode* to *life mode.*

Technical barrier (e.g., a mini-game not working, game "freezing", restart needed, tracking off.)

Change in own/family dynamic (e.g., one parent out of town, schoolwork, sickness)

Feeling arm pain/discomfort after game play


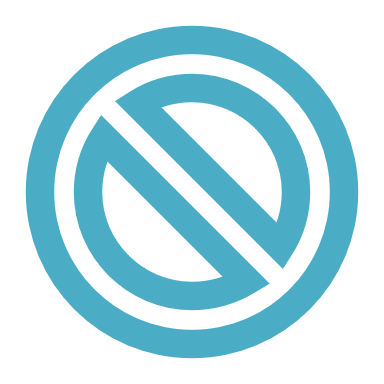


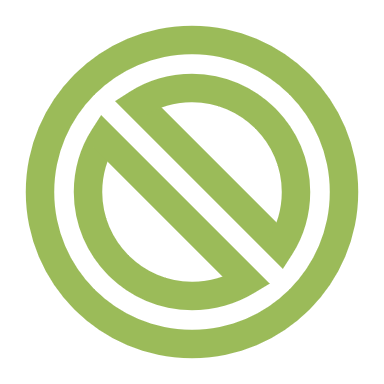


# Table 13. Total playtime (across 12 weeks) per mini-game by participant (Appendix D).

|  | Total play time (minutes across 12-weeks) | | | |
| --- | --- | --- | --- | --- |
| Mini-game | *Marco* | *Alma* | *Ivan* | *Leo* |
| *Wizard’s Adventure* | *113* | *13* | *94* | *131* |
| *Jetpack Bootle* | 48 | *10* | *181* | 101 |
| *Astro Bootle* | 23 | 6 | 52 | *108* |
| *Bootle Paint* | 22 | 0 | 33 | 84 |
| *Bird Adventure* | 45 | 0 | 33 | 21 |
| *Paint Baller* | 39 | 5 | 75 | 58 |
| *Cliff Climber* | 5 | 0 | 5 | 5 |
| *Bootle Kart* | 42 | *11* | 87 | *108* |
| *Bootle Ball* | 53 | 0 | 73 | 30 |
| *Bubble Lab* | *90* | 0 | 38 | 21 |
| *Magic Blocks* | 18 | 4 | 61 | 5 |
| *Colour Fill* | 28 | 9 | *186* | 1 |
| *Bootle Band* | *68* | 4 | 28 | 28 |

*Blue italic text represents the most played mini-games by participant.*

**
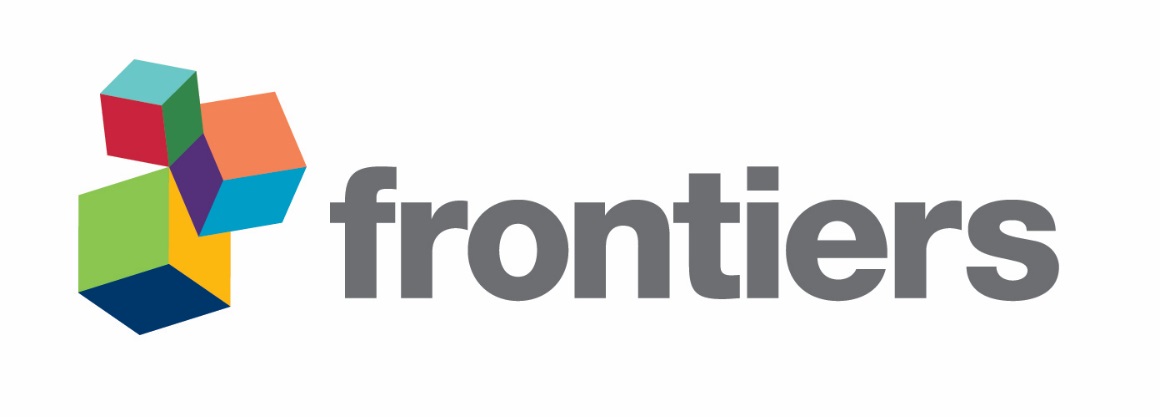
**
